# Radiolytic Gas Production from Aluminum Coupons (Alloy 1100 and 6061) in Helium Environments—Assessing the Extended Storage of Aluminum Clad Spent Nuclear Fuel

**DOI:** 10.3390/ma15207317

**Published:** 2022-10-19

**Authors:** Jacy K. Conrad, Xiaofei Pu, Amey Khanolkar, Trishelle M. Copeland-Johnson, Corey D. Pilgrim, Joseph R. Wilbanks, Elizabeth H. Parker-Quaife, Gregory P. Horne

**Affiliations:** 1 Center for Radiation Chemistry Research, Idaho National Laboratory, P.O. Box 1625, Idaho Falls, ID 83415, USA; 2 Characterization and Post Irradiation Examination, Idaho National Laboratory, P.O. Box 1625, Idaho Falls, ID 83415, USA; 3 Condensed Matter and Materials Physics Group, Idaho National Laboratory, P.O. Box 1625, Idaho Falls, ID 83415, USA; 4 Glenn T. Seaborg Institute, Idaho National Laboratory, P.O. Box 1625, Idaho Falls, ID 83415, USA

**Keywords:** gamma radiolysis, molecular hydrogen generation, aluminum clad spent nuclear fuel

## Abstract

Corrosion of aluminium alloy clad nuclear fuel, during reactor operation and under subsequent wet storage conditions, promotes the formation of aluminium hydroxide and oxyhydroxide layers. These hydrated mineral phases and the chemisorbed and physisorbed waters on their surfaces are susceptible to radiation-induced processes that yield molecular hydrogen gas (H_2_), which has the potential to complicate the long-term storage and disposal of aluminium clad nuclear fuel through flammable and explosive gas mixture formation, alloy embrittlement, and pressurization. Here, we present a systematic study of the radiolytic formation of H_2_ from aluminium alloy 1100 (AA1100) and 6061 (AA6061) coupons in “dry” (~0% relative humidity) and “wet” (50% relative humidity) helium environments. Cobalt-60 gamma irradiation of both aluminium alloy types promoted the formation of H_2_, which increased linearly up to ~2 MGy, and afforded *G*-values of 1.1 ± 0.1 and 2.9 ± 0.1 for “dry” and “wet” AA1100, and 2.7 ± 0.1 and 1.7 ± 0.1 for “dry” and “wet” AA6061. The negative correlation of H_2_ production with relative humidity for AA6061 is in stark contrast to AA1100 and is attributed to differences in the extent of corrosion and varying amounts of adsorbed water in the two alloys, as characterized using optical profilometry, scanning electron microscopy, Raman spectroscopy, and *X*-ray diffraction techniques.

## 1. Introduction

Aluminum-based alloys have been used globally as nuclear research reactor fuel cladding for a wide range of fuel compositions and geometries [1,2]. The combination of radiation, elevated temperature, and water contact—typical in reactor and subsequent wet storage conditions—promotes corrosion processes (e.g., crevice, galvanic, intergranular, and pitting) that impact the integrity and performance of these aluminum-based materials. Consequently, there has been extensive investigation into the thermal and chemical processes responsible for their degradation [1]. Aluminum cladding corrosion is typically initiated when the attendant passivating aluminum oxide layer is compromised, allowing for the formation of additional aluminum oxide (Al_2_O_3_), and/or oxyhydroxide (γ-AlOOH: boehmite) and hydroxide (Al(OH)_3_: bayerite, gibbsite, and/or nordstrandite) layers, which alter the physical and chemical properties of the material. Oxyhydroxides are of particular concern due to their susceptibility to radiolysis and the formation of molecular hydrogen gas (H_2_) [3,4,5,6,7]. The generation of H_2_ can lead to flammable and explosive gas mixtures, alloy embrittlement [8,9,10], and pressurization that can cause additional material stresses [11]. Despite the importance of these radiation-induced H_2_ formation processes, there has been little concerted effort to characterize them under representative conditions, which limits our ability to predict the long-term behaviour of these materials and define appropriate safety margins for their use. This is especially relevant for aluminum-clad spent nuclear fuel (ASNF), of which the United States (U.S.) is currently managing over 18 metric tons [12]. Some of this ASNF is destined for extended (>50 years) dry storage in helium (He) backfilled canisters [13,14]. Validation of this storage strategy is in part dependent on the amount of H_2_ that is radiolytically generated from the cladding within this time.

Aluminum alloys AA1100 and AA6061 make up a large fraction of the U.S. ASNF inventory [12]. Different alloys have different elemental compositions, as given for AA1100 and AA6061 in the Supplementary Materials Table S1. This may influence the structure and morphology of the aluminum corrosion layers, and ultimately affect the radiation response of the system. Although the corrosion susceptibility of AA1100 and AA6061 at elevated temperature (150 °C) and 100% relative humidity (RH) has been investigated systematically to aid in the development of effective strategies for the dry storage of ASNF [15], studies focused on the effect of aluminum alloy composition on H_2_ yield, *G*(H_2_), have not been performed. Comparison between H_2_ production from aluminium alloy types, AA1100 vs. AA6061, highlights potential differences in the relative susceptibility of each alloy’s corrosion layer to radiolysis and H_2_ production.

The radiolytic formation of H_2_ from these oxyhydroxide and hydroxide ‘coated’ aluminum-based materials occurs through a combination of chemisorbed and physisorbed waters, which may undergo radiolysis analogously to bulk water radiolysis [16]:

H_2_O ⇝ H_2_O*, ^•^OH, H^•^, e_aq_^−^, H_2_, H_2_O_2_, H_aq_^+^, (1)

H_2_O* → H_2_ + O, (2)

e_aq_^−^ + e_aq_^−^ (+2H_2_O) → H_2_ + 2OH^−^, (3)

e_aq_^−^ + H_aq_^+^ → H^•^, (4)

e_aq_^−^ + H^•^ + H_2_O → H_2_ + OH^−^, (5)

H^•^ + H_2_O → H_2_ + ^•^OH, (6)

H^•^ + H^•^ → H_2_, (7)

and the potential radiation-induced ionization of the material’s corrosion layers, which generate excitons (h^+^ and e^−^) that propagate its radiolytic degradation via interaction with surface hydroxyl groups (∙∙∙OH) or bound water molecules [17,18,19]:

Al∙∙∙OH ⇝ h^+^ + Al∙∙∙OH^−^, (8)

Al∙∙∙OH^−^ → Al∙∙∙O^•−^ + H^•^, (9)

Al-OH_2_ + h^+^ → Al∙∙∙OH^•^ + H_aq_^+^, (10)

The H^•^ atoms that form can either recombine to re-form the hydroxide group, be trapped in interstitial sites within the corrosion layer, or diffuse through the corrosion layer to combine into H_2_, as per Equation (7), or via H-atom abstraction reactions with chemisorbed and physisorbed waters or aluminum oxide and oxyhydroxide “∙∙∙OH” groups [4,7]. As confirmed with electron paramagnetic resonance (EPR) spectroscopy, the amount of H^•^ atoms trapped in the corrosion layer plateaus as a function of absorbed radiation dose, likely indicating that the trapping sites are saturated [4,7]. Several studies have used EPR to characterize the long-lived “^•^O^−^” group, which remains trapped in the material once the H^•^ atom is removed as H_2_ [4,7,20,21,22,23]. Aside from the removal of H^•^ atoms, gamma irradiation is found to make little difference in the overall structure of the aluminum hydroxide and oxyhydroxide phases [23,24].

The interplay between the radiation-induced changes in the aluminum hydroxide and oxyhydroxides phases and their interaction with adsorbed water molecules influences the yield of H_2_ formed [3,4,5,7,21,23]. For example, powders of the hydroxide phase gibbsite (α-Al(OH)_3_), were found to yield significantly less H_2_ than the oxyhydroxide phase boehmite (γ-AlOOH) [4,5,24], despite the fact that cleavage of an “O-H” bond is more favourable in gibbsite [25]. The difference in H_2_ yield was attributed to the relative ease of diffusion of H^•^ atoms and H_2_ in boehmite when compared to that in gibbsite, allowing more radiolysis products to escape [5,7,21,25]. In addition, diffusion occurs equally in all directions in gibbsite, whereas in boehmite diffusion occurs preferentially at one surface, thus allowing for an increased localized concentration of H^•^ atoms, increasing the probability of radical combination into H_2_ [21,25]. Changes in the H_2_ yield from powders are also seen with RH and particle size [4,5,6,21]. For example, gamma irradiated boehmite powders were found to yield larger volumes of H_2_ for lower amounts of bound water, but no H_2_ for a perfectly dry sample [5,7,21]. This observation was attributed to a strong surface effect, where chemisorbed and physisorbed waters provide a location for the H^•^ atoms to diffuse to, thus preventing their recombination into a “∙∙∙OH” group or confinement in the solid. A monolayer of water molecules on the surface promotes H_2_ production as it concentrates H^•^ atoms, thereby increasing their likelihood of combination or H-atom abstraction from surface-bound waters. This relationship between RH and the production of H_2_ from powdered samples may have significant implications on the expected amounts of H_2_ generated under conditions envisioned for the extended dry storage of ASNF.

In previous work, our group demonstrated that the radiolytic yield—*G*-value—of H_2_ from both “wet” (50% and 100% added RH) and “dry” (~0% RH) corroded aluminum coupons was strongly dependent on the gaseous environment—air, nitrogen (N_2_), and argon (Ar)—present during irradiation [26]. The highest *G*-value was measured for the most chemically inert gaseous environment, Ar, followed by N_2_, and essentially a complete inhibition of H_2_ formation was observed in air. This trend was attributed to a progressive decrease in chemical reactivity towards H_2_ and/or its precursors (e^−^/e_aq_^−^ and H^•^) [16,27]. However, helium (He) has been proposed as the back-fill gas for the extended dry storage of ASNF [13,14]. Although He is also a chemically inert gas, much like Ar, it possesses a far greater first ionization energy than Ar (24.59 eV vs. 15.76 eV, respectively [28]). This difference in first ionization energy may facilitate further chemical change, which may alter the radiolytic yield of H_2_, either by directly ionizing and decomposing H_2_ [29,30,31], or by imparting that ionization energy into the ASNF surface, thereby prompting the formation of additional H_2_. Consequently, understanding the influence of He on the radiolytic behaviour of “wet” and “dry” ASNF is essential for evaluating the feasibility of extended storage of ASNF in He backfilled canisters.

To this end, we report a systematic gamma irradiation study on the impact of He environments on the radiolytic production of H_2_ from corroded aluminum alloy coupons (AA1100 and AA6061) under ambient temperature and ~0% to 50% added RH conditions. Surface characterization techniques were also employed to determine the extent of corrosion and chemical species in the corroded region of the two aluminum alloys. Our results reveal that the composition of the aluminum alloy plays an important role in the radiolytic production of H_2_.

## 2. Materials and Methods

### 2.1. Sample Preparation

AA1100 and AA6061 coupons (2.5 cm × 0.65 cm × 0.15 cm) were purchased from Metals Samples Company—Alabama Specialty Products, Inc. (Munford, AL, USA) Aluminum alloys were selected based on their abundance in the U.S. ASNF inventory [12]. Acetone (HPLC Plus, ≥99.9%) and ethanol (absolute, ≥99.8%) were supplied by Millipore-Sigma (Burlington, MA, USA). Helium was purchased in its highest available purity from Norco Inc. (Boise, ID, USA). Ultra-pure water (18.2 MΩ cm) was used for all water applications.

Aluminium coupon surfaces were polished with 600 grit sandpaper by the manufacturer. Upon receipt, the coupons were cleaned via sequential sonication in acetone, ethanol, and then water, prior to being air dried and weighed. Half of the coupons for each alloy type were subsequently corroded by immersion in water in a temperature-controlled Pyrex water bath for 29 days at 95 °C. Post corrosion, the coupons were recovered, air dried, weighed again, and then transferred into individual specimen bags prior to sample preparation. All aluminium coupons—“pristine” (cleaned only) and corroded (cleaned and corroded)—were subsequently flame-sealed in borosilicate glass ampoules as previously described in reference [26].

### 2.2. Steady-State Gamma Radiolysis

Irradiations were performed using the Idaho National Laboratory (INL) Center for Radiation Chemistry Research (CR2) Foss Therapy Services (Pacoima, CA, USA) Cobalt-60 Irradiator unit. Samples comprised of individually flame-sealed borosilicate glass ampoules containing either a single pristine or corroded AA1100 or AA6061 coupon in a He gaseous environment at either ~0% or 50% added RH—previous work found that >50% added RH led to a decrease in *G*(H_2_) for AA1100 coupons [26], and thus ~0% and 50% were selected as bounding conditions. Samples were loaded into a multi-position sample holder and irradiated at ambient irradiator temperature (~45 °C, as determined using a calibrated NI USB-TC01 Single Channel Temperature Input Device equipped with a K-type thermocouple) over several days or weeks to achieve the desired absorbed gamma dose. Dosimetry was performed for each sample position using Fricke solution [32]. The determined dose rates (95–470 Gy min^−1^) were subsequently corrected for the radioactive decay of cobalt-60 (τ_1/2_ = 5.27 years; E_γ1_ = 1.17 MeV and E_γ2_ = 1.33 MeV) and aluminium metal electron density (0.8673) [26,33].

### 2.3. Headspace Gas Analysis

Quantification of the yield of H_2_ was achieved by gas chromatography (GC) using a Shimadzu Co. (Kyoto, Japan) Nexus GC-2030 gas chromatograph equipped with a thermal conductivity detector (TCD) set at 200 °C. Note, preliminary H_2_ measurements made using a mercuric reduction gas detector struggled with consistent reproducibility [34,35], the TCD did not.

The injection port temperature was 150 °C with a split ratio of 15. The carrier gas was He with a linear velocity of 50.0 cm s^−1^. The column (Restek, #19722 Molecular Sieve 5 Å) had an oven temperature profile of 40 °C for 2 min, followed by a ramp to 50 °C over 20 s, and finally a 1-min hold, for a total run time of 3.33 min per injection. All injections were repeated in triplicate and the results were averaged. This method has an estimated error of ≤10% and limits of detection of 0.01–1% H_2_ at the 95% confidence level. Quality control checks were performed daily to confirm known concentrations of H_2_ relative to measured calibration curves.

A crush-tube method was employed, wherein the flame-sealed ampoules containing the Al coupons were cracked inside a length of tubing (Nalgene, 8005 braided PVC, Thermo Fisher Scientific, Waltham, MA, USA) that was fitted with a septum on one end. The headspace of the tubing was then sampled in 100 µL aliquots with a gas-tight syringe (Hamilton, Giarmata, Romania, Model 1810 RN) and injected into the GC. The ampoule and tubing headspace volumes were determined by filling with water and weighing before and after cracking the ampoules. The pressure in the tubing headspace was calculated iteratively using the ideal gas law with the calculated H_2_ gas yields, the measured headspace volumes for the tubing and the ampoule, and the known pressure of He at which the ampoule was sealed, as outlined in Appendix A. The resulting yields of H_2_ are reported in μmol kg^−1^ of aluminium coupon (base metal and corrosion layer) and as *G*-values—in SI units of μmol J^−1^, were 1 μmol J^−1^ = 9.62 (molecule 100 eV)^−1^—as calculated from the slope of linear fits to the H_2_ data.

### 2.4. Surface Characterization

Surface characterization techniques were employed to identify any correlation between the measured *G*(H_2_) values and the composition of the aluminum oxyhydroxide and hydroxide polymorph surface corrosion species.

The surface roughness of as-received AA1100 and AA6061 coupons was measured using a Keyence Corporation (Osaka, Japan) VK-X250 laser scanning confocal microscope. Surface topography was obtained over multiple 85 µm × 85 µm regions by focusing the laser beam with a 150× microscope objective lens. The average surface roughness was calculated from the surface topography within 15 µm × 15 µm regions between surface relief features introduced by polishing. Representative surface topography maps of as-received AA1100 and AA6061 coupons are shown in Figure S1.

Scanning electron microscopy (SEM) was used to characterize sample morphology and examine the extent of surface corrosion into the bulk specimen by using cross-section cuts. Specimens were characterized using a JEOL (Peabody, MA, USA) JSM 6610 LV equipped with an EDAX (Pleasanton, CA, USA) Apollo X energy-dispersive X-ray spectroscopy (EDS) detector (10 mm^2^ active area). Secondary electron (SE) and backscatter electron (BSE) images were captured at an accelerating voltage of 15 kV and a working distance of ~10 mm.

Raman spectroscopy was used to characterize the localized distribution of surface oxyhydroxide and hydroxide corrosion species initially detected by SEM analysis. Raman spectra were collected in ambient laboratory conditions using a LabRAM HR confocal microscope (HORIBA Jobin Yvon SAS, Edison, NJ, USA) equipped with a long focal length (800 mm) Czerny-Turner type spectrograph. A 532 nm continuous wave laser beam emitted by a Coherent Verdi laser was used for excitation of Raman-active modes. The laser beam was passed through a confocal pinhole of 400 µm diameter, and a motorized slit set to a width of 100 µm, and finally focused at normal incidence on the sample surface to a ~2 µm diameter spot using a 50× objective lens. The power of the laser beam incident on the sample was ~450 µW. Raman spectra were acquired with an exposure time of 20 s and over 2 accumulations in the 20–4000 cm^−1^ range. For each sample, measurements were performed on multiple locations. Processing, analysis, and visualization of the Raman spectra was performed using a user-defined routine in MATLAB (MathWorks Inc., Natick, MA, USA). Details of the signal process methods applied to the measured Raman spectra are provided in the Supplementary Materials. Raman standards used for identification of the corrosion species were obtained through the RRUFF^TM^ database [36] and those reported in the existing literature [24,37].

The X-ray Diffraction (XRD) analysis for each of the reported samples was conducted on a Malvern Panalytical (Malvern, The Netherlands) Empyrean X-ray Diffractometer at the Irradiated Materials Characterization Laboratory (IMCL) in the Materials and Fuels Complex at INL. The XRD analyses were conducted in the Bragg-Brentano geometry, with a Cu Kα X-ray beam at 45 kV and 40 mA, and a PIXEL-3D detector. The sample was loaded onto a zero-background plate to reduce the interference from the background. Because the size of the X-ray beam is larger than the Al coupons, the results from the XRD represent bulk analysis from the entirety of the sample surface. The 2-theta angle ranged from 10 to 90 degrees. The scanning step size was 0.026 degrees, with the counting time of 200 s per step, in continuous scan mode. The data were analysed using HighScore software and compared against the ICDD PDF 4+ 2021 database for phase identification [38]. Rietveld refinement was conducted with the HighScore software to obtain qualitative information on the phase fraction of each sample.

## 3. Results and Discussion

### 3.1. Surface Characterization

**Figure 1:**
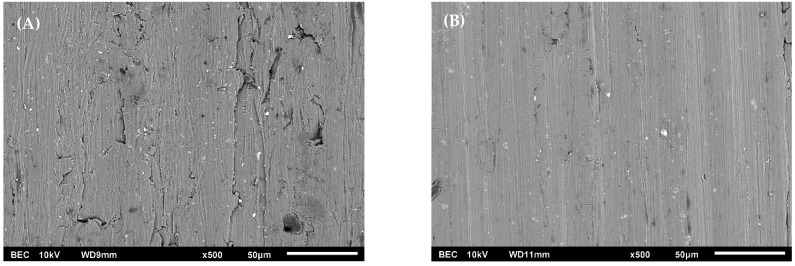
Backscatter electron composition SEM micrograph of the top surface of an as-received coupon of AA1100 (**A**) and AA6061 (**B**), with scale bars of 50 µm.

SEM micrographs of the uncorroded coupon surfaces are shown in Figure 1A,B for AA1100 and AA6061, respectively. Both samples appear somewhat smooth and featureless, aside from residual scratches from the manufacturer polishing process. The surface roughness of an as-received AA1100 coupon was measured to be 0.43 ± 0.110 µm, while that of the AA6061 was 0.14 ± 0.037 µm. The rougher surface of the as-received AA1100 coupons, seen in the form of textured features in the SEM micrograph in Figure 1A, can be attributed to the lower hardness of AA1100 when compared to that of AA6061 [39]. Surface roughness is known to influence the amount of adsorbed water in metals [40]. The relatively higher surface roughness of the pristine AA1100 coupons offers more surface area for water absorption, and thus potentially a greater inventory of H_2_ precursors compared to AA6061.

**Figure 2:**
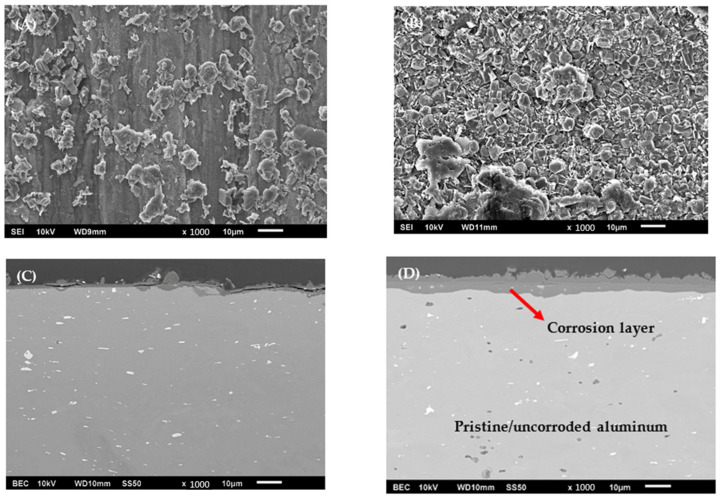
Secondary electron image topographic SEM micrographs of the top surface of a corroded coupon of (**A**) AA1100, and (**B**) AA6061, and backscatter electron composition SEM micrographs the cross-section of (**C**) AA1100, and (**D**) AA6061, with scale bars of 10 µm. The red arrow indicates the corrosion layer.

Corrosion of both alloys resulted in a net weight gain from the formation of hydroxide and oxyhydroxide layers—AA1100 samples gained an average of 1.45 ± 0.16 mg (0.22 ± 0.02%), where AA6061 coupons gained an average of 4.97 ± 0.70 mg (0.81 ± 0.11%) on corrosion. These results would suggest that there was >3× more corrosion in the AA6061 samples than the AA1100 samples, assuming that the two alloys had similar corrosion layer compositions. SEM of the surface and cross-sectioned segments of AA1100 and AA6061 specimens provide some insight into compositional changes at the surface and the extent of corrosion. Figure 2 shows typical BSE SEM micrographs of the top surface and cross-sections of corroded AA1100 (A and C) and AA6061 (B and D) coupons, respectively, qualitatively illustrating compositional differences with respect to image contrast based on the atomic number or Z-contrast. Similar depictions of surface morphology and Z-contrast are observed, suggesting a similar composition of corrosion layers developed on both alloys. Specimens primarily produced distinguishable, equiaxial grains with the occasional large platelets atop and needle features observed subsurface, as shown in Figure S2. These needle-like features have been observed in our previous investigations on corroded AA1100 [26]. Analysis of cross-sectioned AA6061 and AA1100 coupons is illustrated in Figure S3. Note that the uncertainty values in the reported corrosion layer thickness are the standard error of the mean within 95% confidence. AA6061 coupons consistently formed a continuous compact corrosion layer. In contrast, the AA1100 coupons developed a smaller, more sporadic corrosion layer that was less thick than the oxide layer seen in previous work [26]. Comparison of the extent of corrosion through SEM corroborates with the aforementioned weight gain estimate.

**Figure 3:**
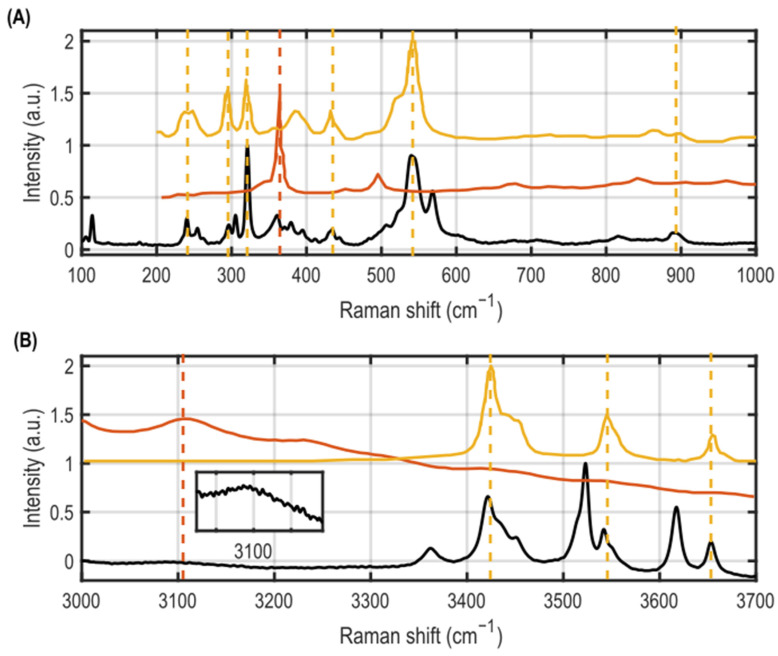
Representative Raman spectrum (black curve) measured on a corroded AA6061 coupon irradiated to a dose of 0.66 MGy in ~0% added RH highlighting peaks in the low (**A**) and high (**B**) wavenumber regimes. Raman spectra from boehmite (orange curve) and bayerite (yellow curve) powders reported previously [24,44] have been overlaid above the measured spectrum to aid in identification of mineral species in the corrosion layer. The orange and yellow dashed vertical lines denote the peak locations in the measured spectra that correspond to matching peak locations in the spectra reported for boehmite and bayerite powders, respectively.

Raman spectroscopy was used to identify the aluminium hydroxide and oxyhydroxide phases formed on the AA1100 and AA6061 coupon surfaces by corrosion, based on examinations of multiple ~2 µm diameter regions per sample. The aluminum oxide/oxyhydroxide mineral phases were identified by comparing peaks in the measured Raman spectra to corresponding peaks in the Raman spectra of alumina phase powders reported previously [24,36,37]. The details of the Raman findings are given in the Supplementary Materials. A representative Raman spectrum measured on a corroded AA6061 coupon that was irradiated to a dose of 0.66 MGy at ~0% added RH is shown in Figure 3. Multiple peaks were observed in the low wavenumber range between 200 to 1000 cm^−1^ (corresponding to the hydroxyl deformation modes), as well as in the high wavenumber region between 3000 and 4000 cm^−1^ (corresponding to the hydroxyl group stretching regime) [37]. Analysis of the measured Raman spectra revealed that bayerite (β-Al(OH)_3_) was the phase most definitively observed as it was seen for all corroded samples of both alloys, alongside peaks consistent with nordstrandite (γ-Al(OH)_3_). The identification of nordstrandite is interesting to note, as it has been suggested that this phase it is formed as an intermediary for transitions between bayerite and gibbsite since it has a crystal structure composed of stacked alternating bayerite and gibbsite layers [41,42,43]. Some peaks consistent with the presence of gibbsite (α-Al(OH)_3_) and boehmite (α-AlO(OH)) were observed in most of the corroded samples, and peaks consistent with corundum (Al_2_O_3_) were observed in a few samples, primarily in the AA1100 coupons. The presence of multiple peaks associated with bayerite and nordstrandite in the measured Raman spectra suggests that these phases reside primarily at the surface (seen in the form of equiaxed crystals in the SEM images). Fewer peaks associated with gibbsite and boehmite were observed in the Raman spectra, suggesting that these species are buried beneath the crystalline surface layer. The sub-surface needle-like features seen in the SEM micrographs may therefore be attributed to the gibbsite and/or boehmite phases. These findings align with the structure observed in our earlier work [26], where oxidation of AA1100 lead to an inner layer of needle-like boehmite with a crystalline outer layer of bayerite. We further note that Raman spectroscopy showed a heterogenous distribution of corrosion products across each sample’s surface, as evidenced by the variation in the intensities of the peaks in the Raman spectra at different locations on the same sample. A representative example of this is shown in the Supplementary Materials (Figure S13).

**Figure 4:**
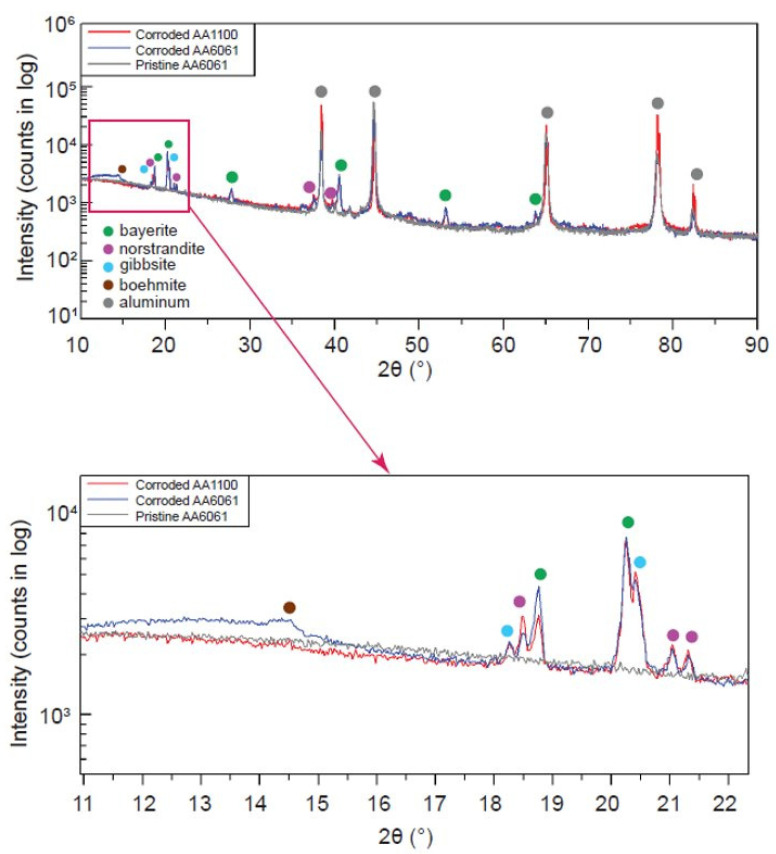
Typical XRD pattern illustrating the major species detected on the surface of a corroded AA6061 coupon.

XRD provides an estimate of the relative phase compositions of the corrosion layers on each alloy. Unlike Raman spectroscopy, which probes a 2 µm spot on the sample surface to a depth of up to 1 µm, [24] XRD scans the entire coupon surface with beam penetration depth up to 100 µm, allowing for prediction of the average composition across the entire corrosion layer. An example of a typical XRD pattern for an unirradiated, corroded AA6061 sample is shown in Figure 4. A summary of the XRD results is given in the Supplementary Materials. While the Raman spectra revealed several peaks associated with bayerite and nordstrandite, suggesting that these phases are primarily surface confined to a 1 µm depth within the corrosion layer, XRD detected a mixture of all three aluminium hydroxide phases (bayerite, nordstrandite, and gibbsite) and a small amount of boehmite (<10%) in the corroded samples of both alloys. Boehmite was more abundant in AA6061. Corundum was seen in the XRD pattern predominantly in the AA1100 samples. In agreement with the weight gain calculations and SEM results, the phase quantification results from the XRD indicate a higher fraction of Al metal in the AA1100 samples in all comparable irradiation and corrosion conditions, indicative of less corrosion in this alloy. Phase quantification resulting from Rietveld refinement of the XRD data suggest that there are no differences in the polymorphic composition of the corrosion layer, supported by the uniform Z-contrast observed in the BSE images during SEM analysis, Figure 2.

The irradiated coupons showed no statistically significant compositional changes by XRD or Raman spectroscopy with applied gamma dose. This observation is consistent with our previous results [26], and findings reported on the irradiation of gibbsite, boehmite, and corundum powders [4,6,24].

### 3.2. H_2_ Yields from Gamma Irradiations

Gamma irradiation of the pristine AA1100 and AA6061 coupons in He environments afforded a linear increase in the yield of H_2_ with the applied dose and were largely independent of the added RH. Figure S4 shows the amount of H_2_ formed radiolytically as a function of dose at 50% added RH for the two pristine aluminum alloy systems. The pristine coupon yields were higher for AA1100 samples than for AA6061 samples, with G(H_2_)_He_ values of (1.23 ± 0.07) and (0.52 ± 0.06) × 10^−4^ µmol J^−1^, respectively. The H_2_ yield from pristine samples is likely to arise simply from the radiolysis of water adsorbed on the coupon surface. This is supported by our surface roughness findings, which indicated that the rougher AA1100 coupons could accommodate more surface adsorbed water than the smoother AA6061 (Figure 1), which explains the higher G(H_2_)_He_ value for AA1100 measured by this work.

**Figure 5:**
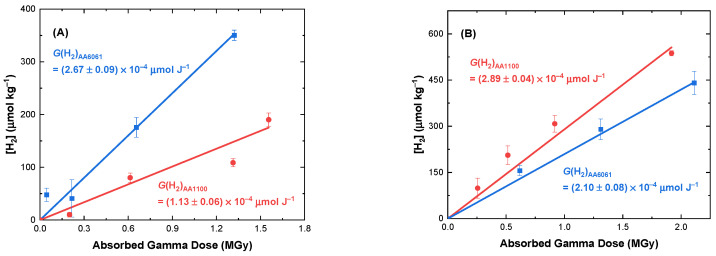
Concentration of H_2_ as a function of absorbed gamma dose from the irradiation of corroded AA1100 (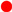) and AA6061 (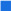) coupons in He environments under ambient temperature and either ~0% (**A**) or 50% (**B**) added RH. Solid lines are linear fits to data, the slopes of which equate to the *G*-value for H_2_ production (µmol J^−1^).

Gamma irradiation of the corroded AA1100 and AA6061 coupons in He environments with ~0% and 50% added RH also resulted in the linear formation of H_2_ with absorbed dose up to ~2 MGy, as shown in Figure 5.

Comparison of *G*(H_2_)_He_ data with the values previously reported for AA1100 irradiated in Ar and N_2_ environments [26], shown in Table 1, demonstrate that He surprisingly has an inhibitory effect on the yield of H_2_, up to 85% lower relative to Ar.

This inhibition of H_2_ production in He is attributed to Penning ionization, whereby radiolytically generated excited states of He (He*) possess energies in excess of the ionization potential of chemical species they collide with, thereby inducing their ionization via de-excitation of He*:

He* + H_2_ → He + H_2_^+^ + e^−^. (11)

This effect has been well characterized for the He*-induced ionization of several noble gases (Ar, krypton, and xenon), other small molecules, such as H_2_, and some metals (e.g., lithium and sodium) [45,46,47,48,49,50,51,52]. Here, Penning ionization of H_2_ likely leads to the decomposition of the resulting molecular hydrogen cation (H_2_^+^) on the aluminium coupon surface:

H_2_^+^ + Al → Al + H^·^ + H^+^. (12)

Although Ar should also be able to induce Penning ionization of H_2_ [29,30,31], the similarity in the first ionization energies of Ar (15.76 eV) and H_2_ (15.4 eV) [28] suggest that this process is less efficient compared to He*-induced ionization of H_2_, as the first ionization for He is 24.59 eV. Overall, these He-inhibition observations are fortuitous for the current strategy for extended dry storage of ASNF in He backfilled canisters.

Although both corroded aluminium alloys yielded H_2_ upon irradiation, their respective *G*(H_2_)_He_ yields and response to changes in RH were different. Overall, the H_2_ yield from AA1100 increased with RH, while that for AA6061 decreased. For ~0% added RH, irradiation of AA6061 coupons afforded larger *G*(H_2_)_He_ values than the corresponding AA1100 coupons, Figure 5A, (2.67 ± 0.09) × 10^−4^ and (1.13 ± 0.06) × 10^−4^ µmol J^−1^ (Table 1). However, upon increasing the added RH to 50%, the inverse trend was observed, i.e., AA6061 yielded a significantly lower yield of H_2_ compared to that from AA1100, Figure 5B, (1.65 ± 0.14) × 10^−4^ vs. (2.90 ± 0.11) × 10^−4^ µmol J^−1^, respectively (Table 1). The radiolytic yield of H_2_ from AA1100 in previous work was found to increase with added RH from ~0% to 50% for both Ar and N_2_ environments, as shown in Table 1, consistent with the behaviour found here for AA1100 irradiated in He environments, where *G*(H_2_)_He_ increased from (1.13 ± 0.1) × 10^−4^ to (2.90 ± 0.1) × 10^−4^ µmol J^−1^ in going from ~0% to 50% added RH. For AA1100, where the corrosion layer is much less extensive, it stands to reason that the H_2_ is primarily produced from adsorbed moisture on the coupon surface. Therefore, an increase in added RH corresponds to an increase in absorbed moisture, which results in a higher yield of H_2_. The ~0% added RH sample coupons were not dried by heating or vacuum after cleaning, nor prior to flame-sealing, therefore, there is likely still some water adhered to the coupon surface. For AA6061, H_2_ is produced by a combination of adsorbed moisture and surface corrosion layer decomposition. Analysis of powder samples have noted that the H_2_ yield per unit mass for aluminium oxides, oxyhydroxides, and hydroxides is larger than the yield from bulk water [5,6,7]. This explains why at ~0% added RH the AA6061 coupons produced a higher yield of H_2_ than the AA1100 coupons. As the % added RH increases, the yield of H_2_ from the aluminium corrosion species will either increase, or decrease, depending on the phase. For example, the yield from boehmite decreases with humidity because the H^•^ atoms generated from the “∙∙∙OH” groups have a decreased likelihood of concentrating on the aluminium oxyhydroxide surface where they can H-abstract from surface-bound waters or combine to form H_2_ [5,7,21]. Due to the complex relationships that the multicomponent corrosion layers on the aluminium alloy surface have with added RH, it is not surprising to see a decrease in the yield for AA6061 with increasing water content. Direct comparisons of the surface areas of the two corroded alloys in future work would provide further insights on the mechanisms of H_2_ generation and removal.

## 4. Conclusions

Gamma irradiation of AA1100 and AA6061 aluminium alloy coupons in “dry” (~0% RH) and “wet” (50% RH) He environments afforded *G*(H_2_)_He_ values lower than those previously measured for complimentary N_2_ and Ar environments. The difference in radiolytic response between the gas environments was attributed to the ability of He to promote Penning ionization, which likely leads to the decomposition of radiolytic H_2_ under the conditions investigated.

Alloy surface morphology between AA1100 and AA6061 was also found to have a significant effect on the radiolytic yield of H_2_ due to differences in the extent of corrosion and the composition of the corrosion layer. AA6061 exhibited a negative correlation in *G*(H_2_)_He_ with added RH, in contrast with AA1100. The difference in radiolytic response between the two alloys was ascribed to changes in the extent of corrosion on the coupon surfaces—namely, AA6061 being significantly more corroded than AA1100, and differences in the H_2_ production with added RH from the aluminium oxide, hydroxide, and oxyhydroxide phases formed.

Overall, the presented *G*(H_2_)_He_ values are important for the development of predictive computer models for evaluating the feasibility of extended storage of ASNF in He backfilled canisters [53].

## Figures and Tables

**Table 1:** Comparison of *G*-values for H_2_ production from the gamma irradiation of corroded AA1100 and AA6061 coupons in He environments, and corresponding AA1100 values in N_2_ and Ar environments, for ~0% and 50% added RH. Ar and N_2_ *G*(H_2_) values were sourced from [26].

Alloy	Gaseous Environment	Added Relative Humidity (%)	*G*(H_2_) (10^−4^ µmol J^−1^)
AA1100	He	~0	1.1 ± 0.1
		50	2.9 ± 0.1
	N_2_	~0	2.0 ± 0.1
		50	4.5 ± 0.1
	Ar	~0	7.3 ± 0.8
		50	9.6 ± 0.6
AA6061	He	~0	2.7 ± 0.1
		50	1.7 ± 0.1

## Data Availability

Data will be made available on request.

## Supplementary Materials

The following supporting information can be downloaded at: https://www.mdpi.com/article/10.3390/ma15207317/s1. Figure S1. Optical micrographs of as-received (a) aluminium alloy 1100 (AA1100) and (b) aluminium alloy 6061 (AA6061) coupons (scale bars represent 20 µm). Representative surface topography maps measured using the Keyence VK-X250 laser scanning confocal microscope for AA1100 and AA6061 coupons are shown in panels (c) and (d), respectively. Figure S2. Backscatter electron composition SEM micrograph of the top surface of a corroded coupon of (a) AA1100, and (b) AA6061, with scale bars of 2 µm, demonstrating the underlying needle-like structure present on both alloys. Figure S3. Backscattered electron images captured from cross-sectioned AA6061 and AA1100 specimens, including estimates of extent of corrosion, including (A) as-received AA6061, and corroded specimens at (B) 0% RH He, maximum dose (7.0 ± 0.3 µm), and (C) 50% RH He, maximum dose (9.7 ± 1.6 µm) versus (D) as-received AA1100, and (E) corroded AA1100 (3.4 ± 0.9 µm). Note that the uncertainty values in the reported corrosion layer thickness are the standard error of the mean within 95% confidence. Figure S4. Concentration of H_2_ as a function of absorbed gamma dose from the irradiation of pristine AA1100 (●) and AA6061 (■) coupons in He under ambient temperature at or 50% RH. Solid lines are linear fits to data, the slopes of which equate to the G-value for H_2_ production. Figure S5. Signal processing procedure applied to a raw Raman spectrum acquired in the 20–1000 cm^−1^ wavenumber range on a corroded AA6061 coupon that received a gamma irradiation dose of 0.66 MGy. Figure S6. Raman spectra in four wavenumber segments (20–1000 cm^−1^, 1000–2000 cm^−1^, 2000–3000 cm^−1^, and 3000–4000 cm^−1^) measured on a pristine (uncorroded) AA1100 coupon that was irradiated to an absorbed dose of 3.47 MGy in a He environment at an added relative humidity of 50%. The peaks observed in the 1000–2000 cm^−1^ and 2000–3000 cm^−1^ range are attributed to a thin passive oxide layer formed on the surface of the aluminium coupon. Figure S7. Comparison of the measured Raman spectra (black curve) in the (left) low and (right) high wavenumber ranges with a Raman spectrum reported on a boehmite powder sample. Figure S8. Comparison of the measured Raman spectra (black curve) in the (left) low and (right) high wavenumber ranges with a Raman spectrum reported on a bayerite powder sample. Figure S9. Comparison of the measured Raman spectra (black curve) in the (left) low and (right) high wavenumber ranges with a Raman spectrum reported on a gibbsite powder. Figure S10. Comparison of the measured Raman spectra (black curve) in the (left) low and (right) high wavenumber ranges with a Raman spectrum reported on a diaspore sample. Figure S11. Comparison of the measured Raman spectra (black curve) in the (left) low and (right) high wavenumber ranges with a Raman spectrum reported on a nordstrandite sample. Figure S12. Comparison of the measured Raman spectra (black curve) in the low wavenumber ranges with a Raman spectrum reported on a corundum sample. Figure S13. Spot-to-spot variations in the peak intensities of representative Raman spectra acquired at multiple locations of an aluminium alloy 1100 sample that was pre-corroded and irradiated to a dose of 0.05 MGy in a ~0% RH environment. The top panel illustrates location-dependent variations in the Raman peak features in the low wave number range, while the bottom panel illustrates similar features in the high wave number range. Table S1: Elemental composition (wt %) for the two alloys studied. Table S2: Summary of mineral phases identified from Raman spectra on AA1100 coupons. Table S3: Summary of mineral phases identified from Raman spectra on AA6061 coupons. Table S4: Phase fractions obtained from Rietveld refinement of XRD results. References [24,36,37,54] are cited in supplementary materials.

## Appendix A. H_2_ Gas Yield Calculations

The GC calibration curve was generated in terms of the percentage of H_2_ present in 100 µL of injected gas. To calculate the number of moles of H_2_ produced in the ampoule, $n_{H_{2}}$, the ideal gas law was used:

(A1) $$n_{H_{2}}=\frac{X_{H_{2}}p_{\mathrm{total}}V_{\mathrm{total}}}{RT},$$

where $X_{H_{2}}$ is the mole fraction of H_2_ determined from GC, $V_{\mathrm{total}}$ and $p_{\mathrm{total}}$ are the total tubing headspace volume and the pressure once the ampoule is cracked, *R* is the gas constant, and *T* is the temperature in Kelvin.

The pressure in the tubing once the ampoule is cracked was calculated using the ideal gas law via:

(A2) $$p_{\mathrm{total}}V_{\mathrm{total}}=\left(p_{\mathrm{tube}}V_{\mathrm{tube}}+p_{\mathrm{amp}}V_{\mathrm{amp}}+RTn_{H_{2}}\right)$$

where the subscripts “tube” and “amp” indicate the pressure, *p*, and volume, *V*, of the tubing and the ampule, respectively, prior to cracking the ampoule. These headspace volumes are determined by filling with water and weighing before and after cracking the ampoules. The initial pressure of the tubing is atmospheric (*p*_tube_ = 1.01 bar), and the ampoule pressure before H_2_ generation is known from the pressure of He used to backfill the evacuated ampoules when flame sealing (*p*_amp_ = 0.843 bar).

Equations (A1) and (A2) are solved iteratively to ultimately yield the number of moles of H_2_. This value was then divided by the aluminum coupon mass (base metal and corrosion layer) to obtain gas yields in µmol kg^−1^.
