# Supplementary material for: Radiolytic Gas Production from Aluminum Coupons (Alloy 1100 and 6061) in Helium Environments—Assessing the Extended Storage of Aluminum Clad Spent Nuclear Fuel
Source: Materials (Basel). 2022 Oct 19;15(20):7317. doi: 10.3390/ma15207317 (PMC9607483; doi:10.3390/ma15207317)
Supplement: Supplementary file 1 [file materials-15-07317-s001.zip › materials-1917290-supplementary.pdf]

materials

MDPI

Supplementary Materials

Radiolytic Gas Production from Aluminium Coupons (Alloy 1100 and 6061) in Helium Environments – Assessing the Extended Storage of Aluminium Clad Spent Nuclear Fuel

Jacy K. Conrad [1,*], Xiaofei Pu [2], Amey Khanolkar [3], Trishelle M. Copeland-Johnson [2,4], Corey D. Pilgrim [1], Joseph R. Wilbanks [1], Elizabeth H. Parker-Quaife [1] and Gregory P. Horne [1,*]

[1] Center for Radiation Chemistry Research, Idaho National Laboratory, P.O. Box 1625, Idaho Falls, ID 83415, USA; corey.pilgrim@inl.gov (C.D.P.); joseph.wilbanks@inl.gov (J.R.W.); eparkerquaife@gmail.com (E.H.P.-Q.)
[2] Characterization and Post Irradiation Examination, Idaho National Laboratory, P.O. Box 1625, Idaho Falls, ID 83415, USA; xiaofei.pu@inl.gov (X.P.); trishelle.copelandjohnson@inl.gov (T.M.C.-J.)
[3] Condensed Matter and Materials Physics Group, Idaho National Laboratory, P.O. Box 1625, Idaho Falls, ID 83415, USA; amey.khanolkar@inl.gov
[4] Glenn T. Seaborg Institute, Idaho National Laboratory, P.O. Box 1625, Idaho Falls, ID 83415, USA
* Correspondence: jacy.conrad@inl.gov (J.K.C.); gregory.horne@inl.gov (G.P.H.)

Citation: Conrad, J.K.; Pu, X.; Khanolkar, A.; Copeland-Johnson, T.M.; Pilgrim, C.D.; Wilbanks, J.R.; Parker-Quaife, E.H.; Horne, G.P. Radiolytic Gas Production from Aluminum Coupons (Alloy 1100 and 6061) in Helium Environments—Assessing the Extended Storage of Aluminum Clad Spent Nuclear Fuel. *Materials* **2022**, *15*, 7317. https://doi.org/10.3390/ma15207317

Academic Editor: Scott M. Thompson

Received: 30 August 2022
Accepted: 12 October 2022
Published: 19 October 2022

Publisher's Note: MDPI stays neutral with regard to jurisdictional claims in published maps and institutional affiliations.

Copyright: © 2022 by the authors. Licensee MDPI, Basel, Switzerland. This article is an open access article distributed under the terms and conditions of the Creative Commons Attribution (CC BY) license (https://creativecommons.org/licenses/by/4.0/).

Elemental composition of AA1100 and AA6061

Table S1. Elemental composition (wt %) for the two alloys studied, from Reference [1].

Alloy	Al	Zn	Mn	Be	Cu	Fe	Si	Ti	Mg	Cr
AA1100	99	0.1	0.05	0.0008	0.1	0.95	-	-	-	-
AA6061	95.8	0.25	0.15	-	0.15	0.7	0.8	0.15	0.8	0.1

Surface roughness measurements of as-received coupons

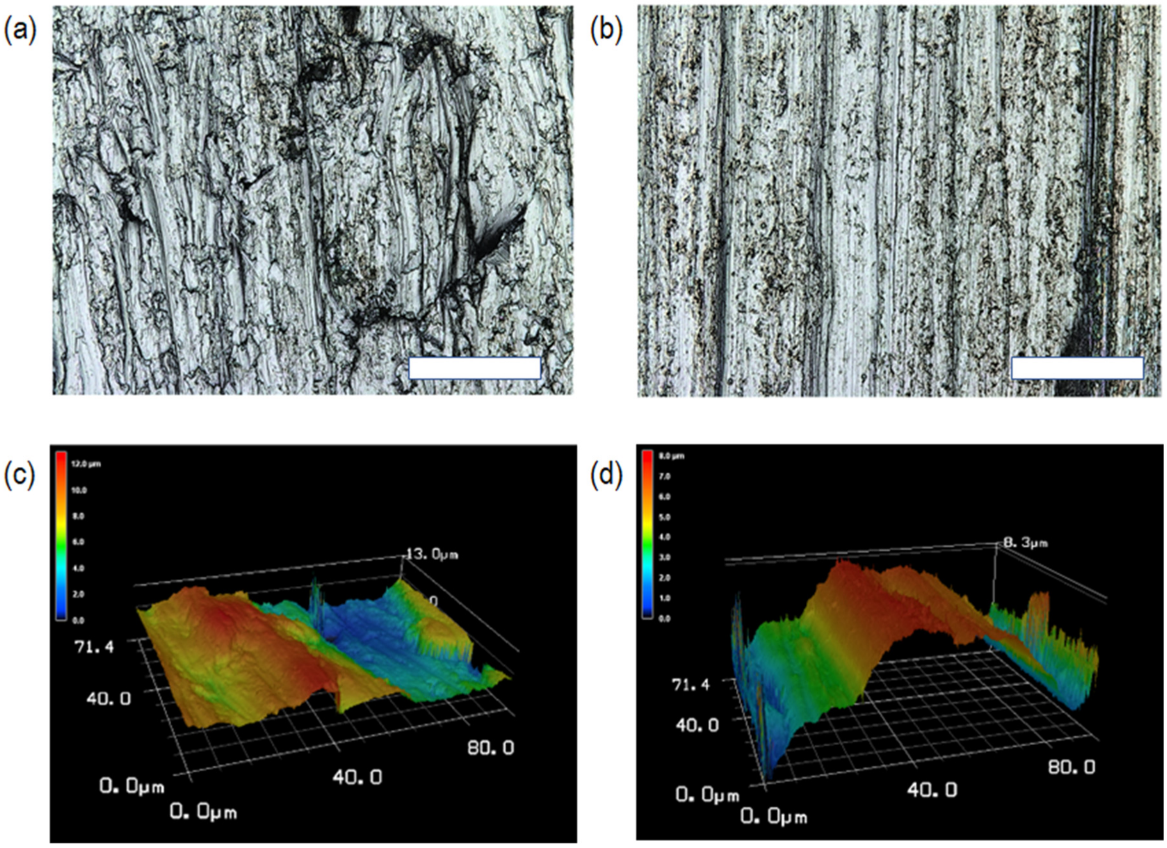

Figure S1. Optical micrographs of as-received (a) aluminium alloy 1100 (AA1100) and (b) aluminium alloy 6061 (AA6061) coupons (scale bars represent 20 µm). Representative surface topography maps measured using the Keyence VK-X250 laser scanning confocal microscope for AA1100 and AA6061 coupons are shown in panels (c) and (d), respectively. .

SEM

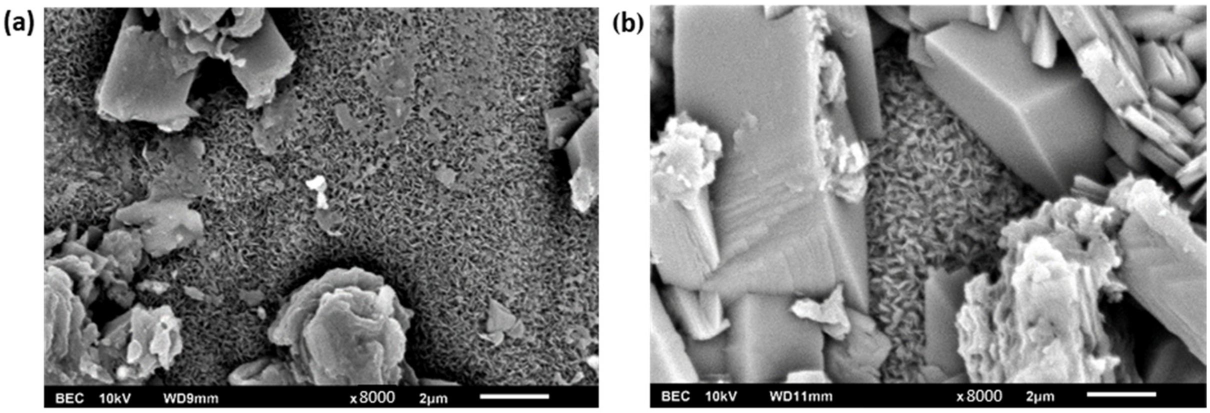

Figure S2. Backscatter electron composition SEM micrograph of the top surface of a corroded coupon of **(a)** AA1100, and **(b)** AA6061, with scale bars of 2 µm, demonstrating the underlying needle-like structure present on both alloys.

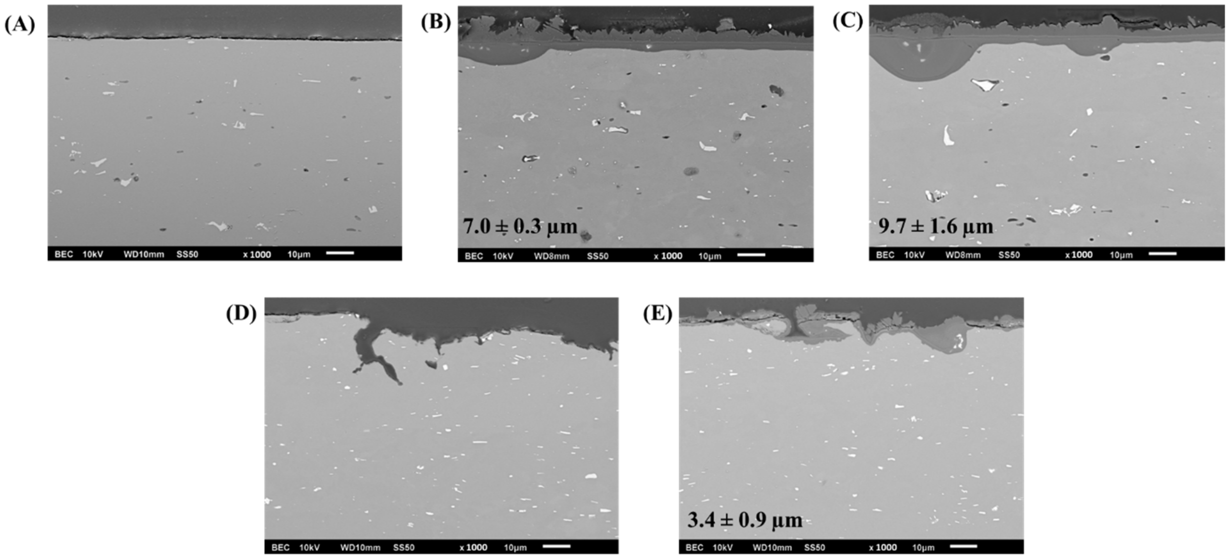

Figure S3. Backscattered electron images captured from cross-sectioned AA6061 and AA1100 specimens, including estimates of extent of corrosion, including (A) as-received AA6061, and corroded specimens at (B) 0% RH He, maximum dose (7.0 ± 0.3 µm), and (C) 50% RH He, maximum dose (9.7 ± 1.6 µm) versus (D) as-received AA1100, and (E) corroded AA1100 (3.4 ± 0.9 µm). Note that the uncertainty values in the reported corrosion layer thickness are the standard error of the mean within 95% confidence.

H_2 Yields for Pristine Coupons

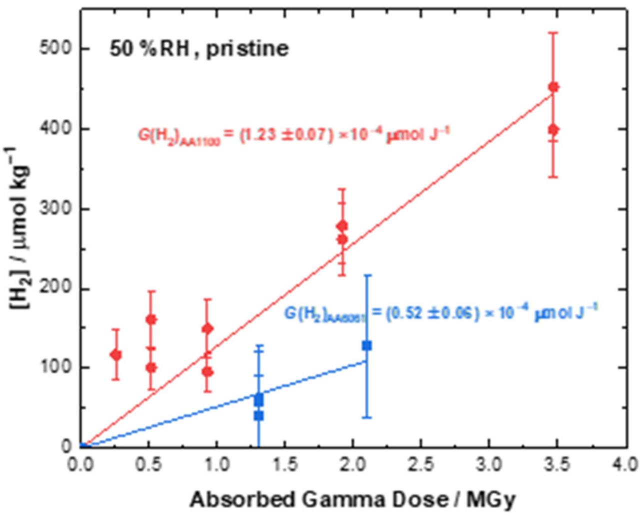

Figure S4. Concentration of H_2 as a function of absorbed gamma dose from the irradiation of pristine AA1100 (●) and AA6061 (■) coupons in He under ambient temperature at or 50% RH. Solid lines are linear fits to data, the slopes of which equate to the *G*-value for H_2 production.

Raman spectroscopy

Raman spectra were acquired on pristine and corroded aluminium alloy 1100 (AA1100) and 6061 (AA6061) coupons, both in unirradiated and irradiated conditions. Spectra were acquired in the wavenumber range of 20 – 4000 cm^{-1}, in four segments (20 – 1000 cm^{-1}, 1000 – 2000 cm^{-1}, 2000 – 3000 cm^{-1}, and 3000 – 4000 cm^{-1}). Prior to analysis of the peaks in the measured Raman spectra, the following signal processing routine was applied in MATLAB®. A smoothing function in MATLAB® that uses a five-point moving

average and a Loess quadratic-fit filter was applied to the raw spectrum to reduce high-frequency noise. A linear fit was applied and consequently subtracted from the filtered spectrum to remove any low-frequency background. The amplitude of the subtracted signal was then normalized. This procedure ensured that the peaks in the Raman spectrum were clearly discerned and not obscured by the background in the signal. Figure S5 illustrates the signal processing procedure applied to a raw Raman spectrum measured in the 20 – 1000 cm^{-1} wavenumber range on a corroded AA6061 coupon that received a gamma radiation dose of 0.66 MGy.

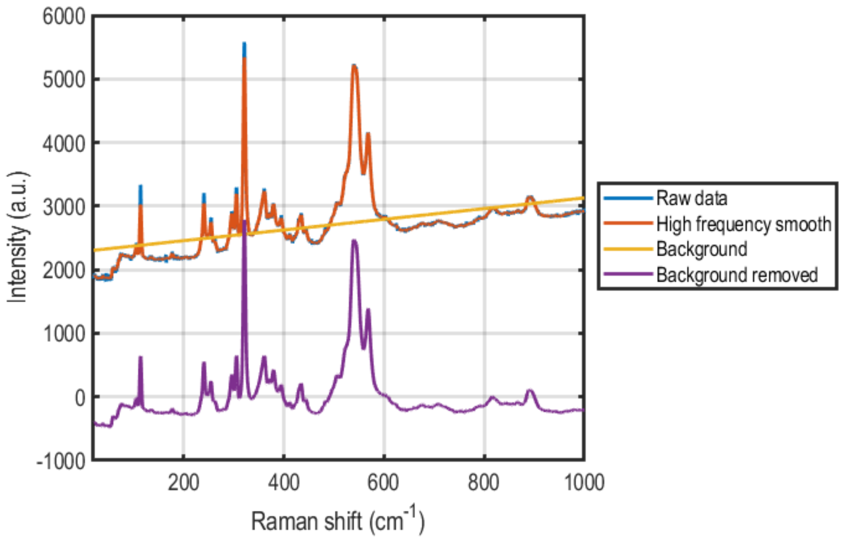

Figure S5. Signal processing procedure applied to a raw Raman spectrum acquired in the 20 – 1000 cm^{-1} wavenumber range on a corroded AA6061 coupon that received a gamma irradiation dose of 0.66 MGy.

Figure S6 shows the Raman spectra in four wavenumber segments (20 – 1000 cm^{-1}, 1000 – 2000 cm^{-1}, 2000 – 3000 cm^{-1}, and 3000 – 4000 cm^{-1}) measured on a pristine (uncorroded) AA1100 coupon that was irradiated to a dose of 3.47 MGy in a helium (He) environment at an added relative humidity (RH) of 50%. The spectra did not reveal any peaks in the 20 – 1000 cm^{-1} and 3000 – 4000 cm^{-1}. Distinct peaks were however observed in the 1000 – 2000 cm^{-1} and 2000 – 3000 cm^{-1} wavenumber range. Since aluminium metal does not exhibit any Raman-active modes, the observed peaks were attributed to originate from a nanometric thin passive oxide layer formed on the surface of the aluminium coupon.

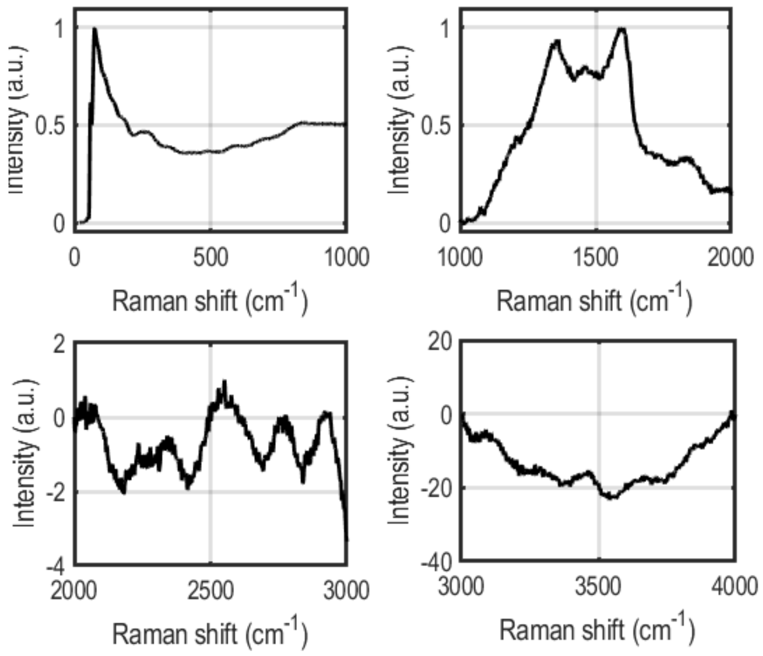

Figure S6. Raman spectra in four wavenumber segments (20 – 1000 cm^{-1}, 1000 – 2000 cm^{-1}, 2000 – 3000 cm^{-1}, and 3000 – 4000 cm^{-1}) measured on a pristine (uncorroded) AA1100 coupon that was irradiated to an absorbed dose of 3.47 MGy in a He environment at an added relative humidity of 50%. The peaks observed in the 1000 – 2000 cm^{-1} and 2000 – 3000 cm^{-1} range are attributed to a thin passive oxide layer formed on the surface of the aluminium coupon.

Similar peaks in the 1000 – 2000 cm^{-1} and 2000 – 3000 cm^{-1} wavenumber range were also seen in the pristine AA6061 coupons. The corroded AA1100 and AA6061 coupons, however, exhibited drastically different Raman spectra compared to their pristine counterparts. In all the corroded coupons, distinct peaks were observed in the 20 – 1000 cm^{-1} and 3000 – 4000 cm^{-1} wavenumber regions. Raman peaks have been reported in alumina mineral phases such as gibbsite, bayerite, diaspore, boehmite, nordstrandite, and corundum in the same wavenumber regions [2]. We compared our measured Raman spectra to those reported from powders of aluminium oxide/oxyhydroxide mineral phases in the RRUFF database [3] and in the literature [2,4] to identify the presence of the particular mineral species in the corrosion layer.

Figure S7 shows a comparison of Raman spectrum measured on a corroded and unirradiated AA1100 coupon (shown with the black curve) and a corresponding spectrum measured on Boehmite powder (blue curve) as reported in reference [4]. An agreement with 1 peak in the low wavenumber range (20 – 1000 cm^{-1}) and with 2 peaks in the high wavenumber range (3000 – 4000 cm^{-1}) is seen, indicating a moderate quantity of the boehmite phase in the region interrogated by the Raman laser.

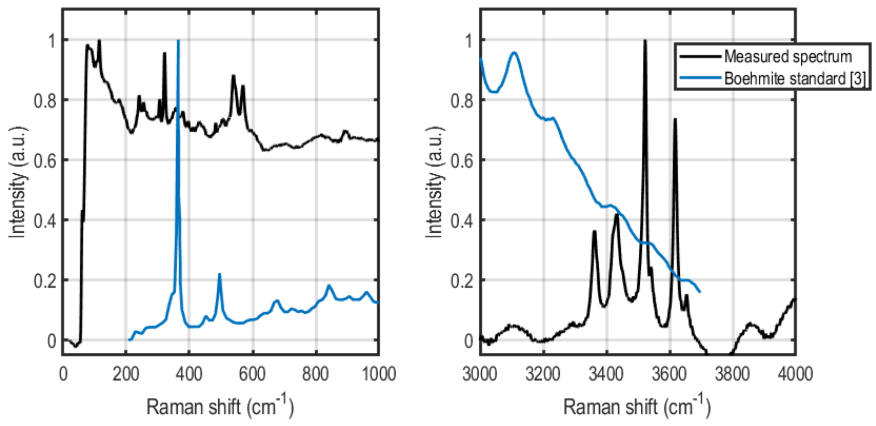

Figure S7. Comparison of the measured Raman spectra (black curve) in the (left) low and (right) high wavenumber ranges with a Raman spectrum reported on a boehmite powder sample in reference [3].

A comparison of the Raman spectrum measured on the same AA1100 coupon with the corresponding spectrum acquired on the bayerite mineral phase is shown in Figure S8. Excellent agreement with at least three peaks in the low and high wavenumber regions suggests an abundant presence of bayerite in the interrogated area.

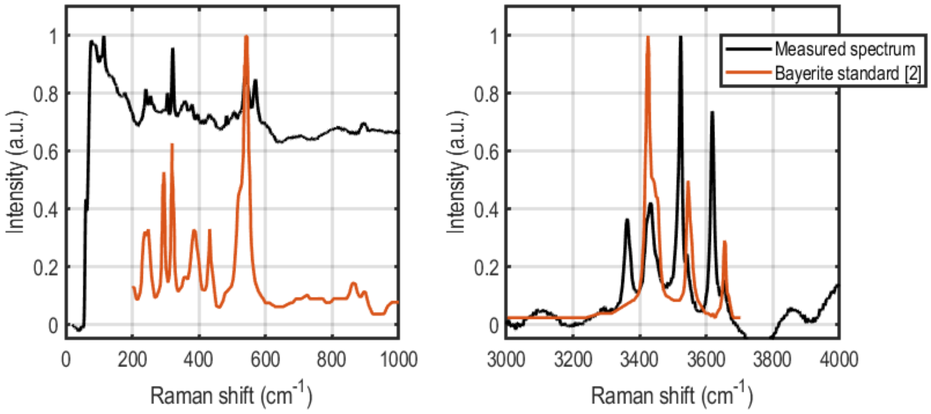

Figure S8. Comparison of the measured Raman spectra (black curve) in the (left) low and (right) high wavenumber ranges with a Raman spectrum reported on a bayerite powder sample in reference [2].

A comparison of the same Raman spectrum measured with the corresponding spectrum acquired on the gibbsite mineral phase is shown in Figure S9. Again, excellent agreement with at least three peaks in the low and high wavenumber regions suggests an abundant presence of gibbsite in the interrogated area.

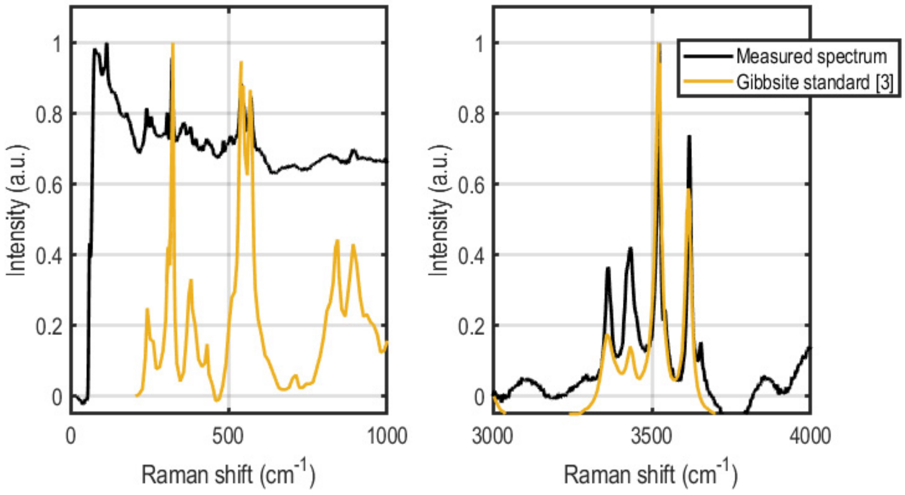

Figure S9. Comparison of the measured Raman spectra (black curve) in the (left) low and (right) high wavenumber ranges with a Raman spectrum reported on a gibbsite powder sample in reference [3].

Figure S10 shows a comparison of our measured Raman spectrum on the same sample with a reference spectrum for diaspore. We see that there are no matching peaks between the two spectra.

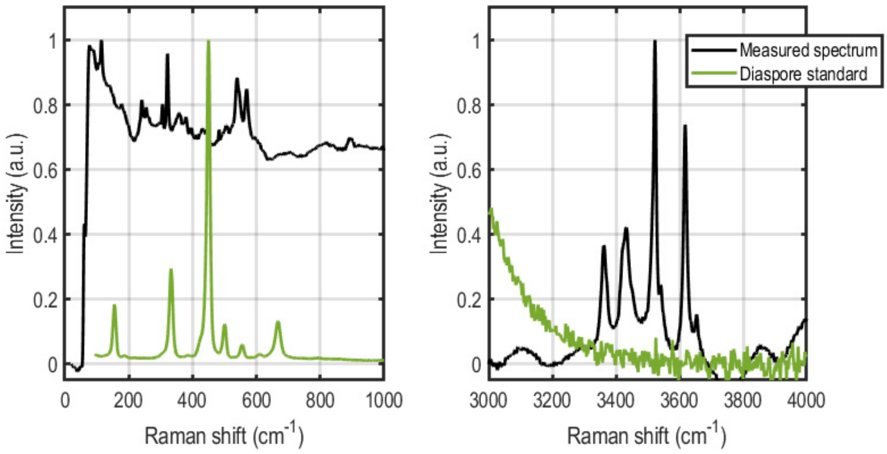

Figure S10. Comparison of the measured Raman spectra (black curve) in the (left) low and (right) high wavenumber ranges with a Raman spectrum reported on a diaspore sample in reference [2].

Figure S11 shows a comparison of our measured Raman spectrum on the same sample with a reference spectrum for nordstrandite. We see that agreement with at least three peaks in both low and high wavenumber ranges, indicating an abundant quantity of the nordstrandite phase in the measured area.

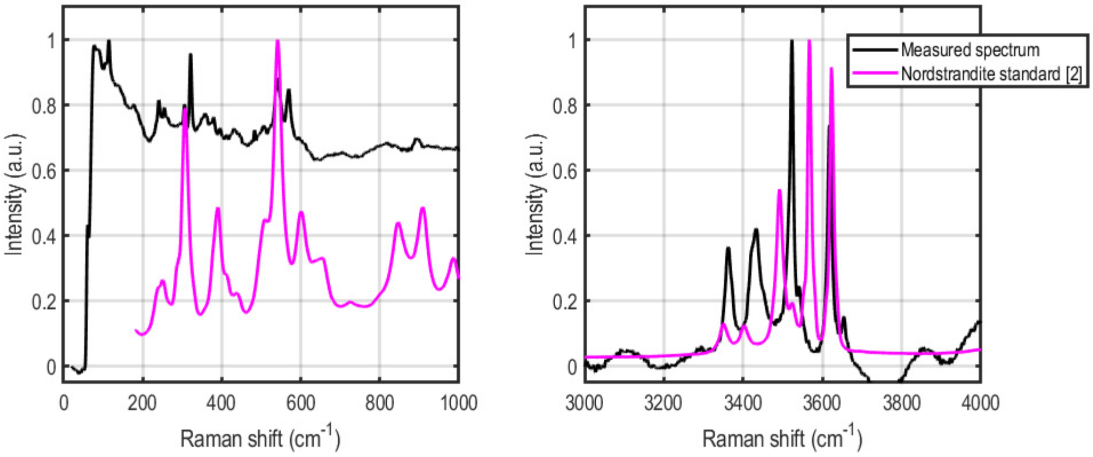

Figure S11. Comparison of the measured Raman spectra (black curve) in the (left) low and (right) high wavenumber ranges with a Raman spectrum reported on a nordstrandite sample in reference [2].

Finally, a comparison of the measured Raman spectrum with that for the corundum phase (available only in the low wavenumber range) showed agreement with only 1 peak, indicating a moderate presence of the phase in the measured region.

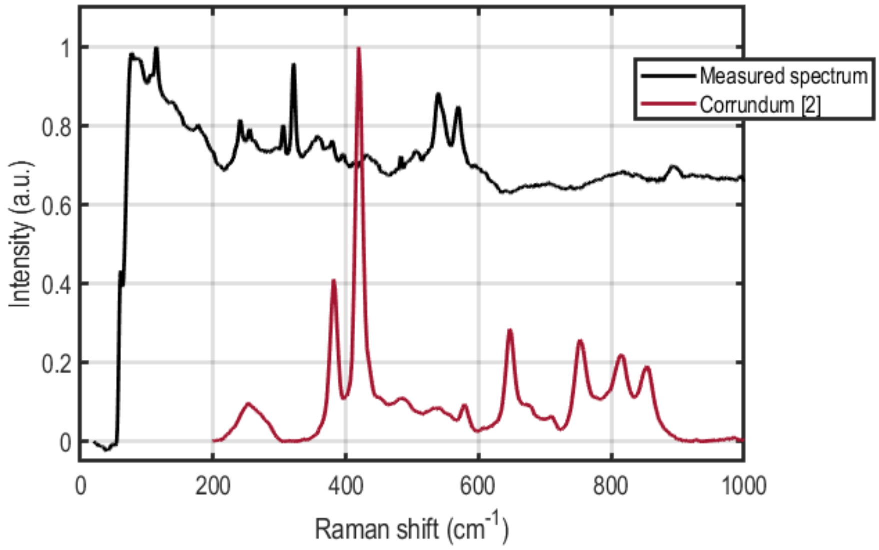

Figure S12. Comparison of the measured Raman spectra (black curve) in the low wavenumber ranges with a Raman spectrum reported on a corundum sample in reference [2].

We also note variations in the peak intensities in Raman spectra acquired at different locations within the same sample were also observed. These variations are indicative of a heterogeneous distribution of corrosion species on the surface of the sample. A representative example of such variations is shown in Figure S13.

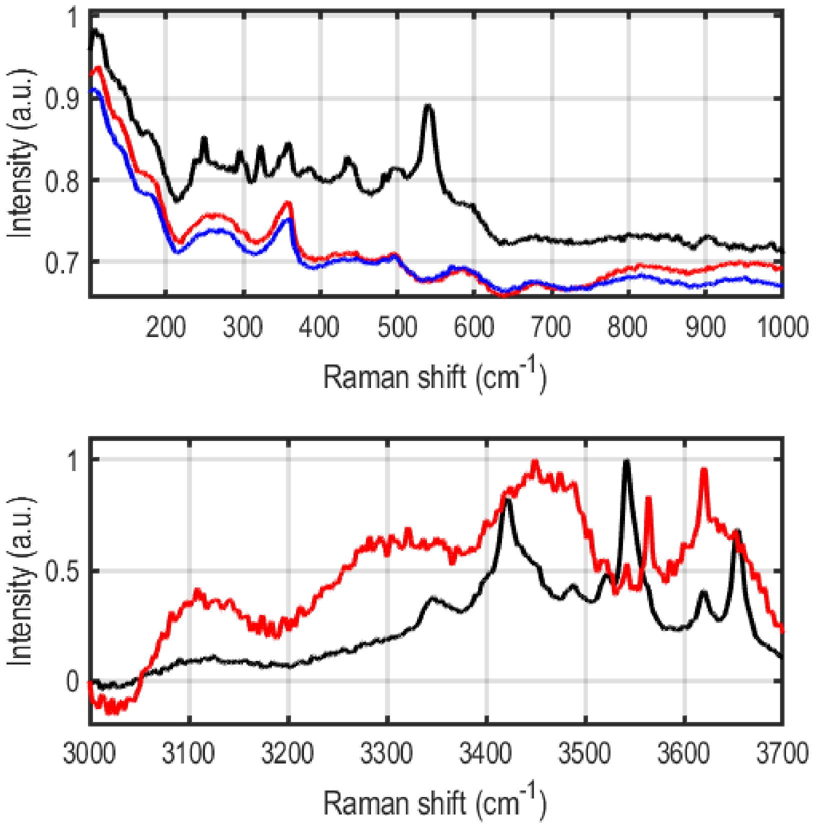

Figure S13. Spot-to-spot variations in the peak intensities of representative Raman spectra acquired at multiple locations of an aluminium alloy 1100 sample that was pre-corroded and irradiated to a dose of 0.05 MGy in a 0% RH environment.

The top panel illustrates location-dependent variations in the Raman peak features in the low wave number range, while the bottom panel illustrates similar features in the high wave number range.

Based on the qualitative comparison of peaks, the relative abundance of a particular aluminium oxide/oxyhydroxide mineral phase in the corrosion layer was determined from the number of peaks in our measured spectrum that lined up with those in the spectra reported in the literature for each phase. An agreement of three or more peaks indicated an abundant quantity of the mineral phase, and is indicated by the cells in green in the following tables. Yellow cells indicate that one or two peaks associated with the aluminium oxyhydroxide mineral phase were detected in the measured Raman spectra. Finally, red cells indicate that no peaks associated with the aluminium oxyhydroxide mineral phase were detected in the measured Raman spectra

Table S2. Summary of mineral phases identified from Raman spectra on AA1100 coupons.

Condition	RH% He	Gamma Dose (MGy)	Bayerite	Nordstandite	Boehmite	Gibbsite	Corrundum	Diaspore
Pristine	50%	3.47						
Pristine	50%	1.92						
Pristine	100%	0.22						
Pristine	100%	0.92						
Pristine	100%	5.07						
Pristine	100%	5.07						
Corroded	Blank	No dose						
Corroded	Blank	No dose						
Corroded	Blank	No dose						
Corroded	0%	0.05						
Corroded	0%	1.31						
Corroded	50%	1.92						
Corroded	50%	1.92						
Corroded	50%	3.47						
Corroded	50%	3.47						
Corroded	100%	0.25						
Corroded	100%	0.92						
Corroded	100%	5.07						

Table S3. Summary of mineral phases identified from Raman spectra on AA6061 coupons.

Condition	RH% He	Gamma Dose (MGy)	Bayerite	Nordstandite	Boehmite	Gibbsite	Corrundum	Diaspore
Pristine	0%	No dose						
Pristine	0%	1.32						
Pristine	0%	1.32						
Pristine	0%	0.04						
Pristine	50%	2.1						
Pristine	50%	0.05						
Corroded	Blank	No dose						
Corroded	Blank	No dose						
Corroded	0%	0.04						
Corroded	0%	0.66						
Corroded	0%	0.66						
Corroded	0%	1.32						
Corroded	0%	1.32						
Corroded	50%	0						
Corroded	50%	0.22						
Corroded	50%	0.22						
Corroded	50%	2.11						
Corroded	50%	2.11						
Corroded	50%	2.11						

X-ray Diffraction (XRD)

Rietveld refinement was performed on selected **corroded** XRD samples for both AA1100 and AA6061 coupons with different corrosion conditions, as listed in Table S3.

Table S4. Phase fractions obtained from Rietveld refinement of XRD results.

Phase fraction from Rietveld Refinement							
**sample**	**condition**	**Al**	**Gibbs-ite**	**Bayer-ite**	**Nordstrandite**	**Boehmite**	**Corundum**
AA1100	No dose	92.5%	1.6%	0.0%	1.8%	1.6%	2.4%
	No dose	82.6%	0.0%	0.8%	9.3%	3.6%	3.8%
	0% RH 0.05MGy	97.4%	0.0%	0.0%	1.2%	1.4%	0.0%
	0% RH 1.3-1.5MGy	94.7%	1.0%	0.0%	1.5%	1.3%	1.5%
	50% RH 1.92MGy	77.1%	2.9%	13.7%	6.0%	0.3%	0.0%
	50%RH 3.5MGy	75.6%	3.4%	14.8%	6.2%	0.0%	0.0%
AA6061	No dose	55.2%	5.3%	23.9%	14.4%	1.2%	0.0%
	0%RH 0.04MGy	58.9%	3.3%	22.7%	13.7%	1.4%	0.0%
	0%RH 1.3MGy	54.6%	3.1%	21.6%	19.2%	1.5%	0.0%
	0%RH 1.3MGy	57.4%	3.6%	22.2%	15.3%	1.5%	0.0%
	50% RH 0MGy	53.8%	6.1%	16.7%	22.7%	0.7%	0.0%
	50% RH 2MGy	53.5%	1.6%	26.1%	18.5%	0.3%	0.0%

Phase fraction among the non-Al phases						
**sample**	**condition**	**Gibbsite**	**Bayerite**	**Nordstrandite**	**Boehmite**	**Corundum**
AA1100	No dose	21%	0%	24%	21%	32%
	No dose	0%	5%	53%	21%	22%
	0% RH 0.05MGy	0%	0%	46%	54%	0%
	0% RH 1.3-1.5MGy	19%	0%	28%	25%	28%
	50% RH 1.92MGy	13%	60%	26%	1%	0%
	50%RH 3.5MGy	14%	61%	25%	0%	0%
AA6061	No dose	12%	53%	32%	3%	0%
	0%RH 0.04MGy	8%	55%	33%	3%	0%
	0%RH 1.3MGy	7%	48%	42%	3%	0%
	0%RH 1.3MGy	8%	52%	36%	4%	0%
	50% RH 0MGy	13%	36%	49%	2%	0%
	50% RH 2MGy	3%	56%	40%	1%	0%

References

1. Anggono, A. D., Riyadi, T., Sarjito, S., Triyoko, D., Sugito, B., & Hariyanto, A., 2018. Influence of tool rotation and welding speed on the friction stir welding of AA 1100 and AA 6061-T6. *AIP Conf. Proc.* **1977**, *020054*. doi:10.1063/1.5042910.
2. Ruan, H.D., Frost, R.L. and Kloprogge, J.T., 2001. Comparison of Raman spectra in characterizing gibbsite, bayerite, diaspore and boehmite. *J. Raman Spectrosc.* **2001**, *32*, 745–750.
3. Lafuente B.; Downs R.T.; Yang H.; Stone N The power of databases: the RRUFF project. In *Highlights in Mineralogical Crystallography*, Armbruster T.; Danisi R.M. Eds.; W. De Gruyter: Berlin, Germany, **2015**, 1-30.
4. Huestis, P., Pearce, C.I., Zhang, X., N'Diaye, A.T., Rosso, K.M. and LaVerne, J.A., 2018. Radiolytic stability of gibbsite and boehmite with adsorbed water. *J. Nucl. Mater.* **2018**, *501*, 224–233.
